# Prolonged Ileus in an Infant Presenting with Primary Congenital Hypothyroidism

**DOI:** 10.1155/2015/584735

**Published:** 2015-03-17

**Authors:** Caroline Chua, Shilpa Gurnurkar, Yahdira Rodriguez-Prado, Victoria Niklas

**Affiliations:** ^1^Division of Neonatology, Department of Pediatrics, University of Central Florida College of Medicine, Nemours Children's Hospital, Orlando, FL 32827, USA; ^2^Division of Endocrinology, Department of Pediatrics, University of Central Florida College of Medicine, Nemours Children's Hospital, Orlando, FL 32827, USA; ^3^Division of Neonatology and Newborn Services, Olive View UCLA Medical Center, Los Angeles, CA 91342, USA

## Abstract

Congenital hypothyroidism (CH) is the most common endocrine disorder affecting the newborn. Universal newborn screening (NBS) has virtually eliminated the static encephalopathy and devastating neurodevelopmental syndrome known as cretinism. This report describes the presentation of an infant referred by the primary pediatrician to our hospital at 12 days of age for confirmatory testing after the NBS was consistent with CH. The infant had hypoglycemia secondary to lethargy and poor feeding and required transfer to the neonatal intensive care unit for worsening abdominal distension despite normalization of serum thyroid function tests following hormone replacement. In particular, the recalcitrant ileus and secondary bowel obstruction resulted in an additional diagnostic workup and lengthened hospital day. Our report highlights the acute gastrointestinal consequences of hypothyroidism despite evidence of effective treatment. We believe that the preclinical detection and immediate therapy for CH have lessened the prevalence of this presentation in general practice, and hence practitioners are less likely to be familiar with its natural history and management.

## 1. Introduction

Congenital hypothyroidism (CH) is the most common endocrine disorder presenting in the newborn period with a prevalence of approximately 1 in 2500 births [1]. The failure to recognize and treat CH may result in static encephalopathy and neurodevelopmental disability, known historically as cretinism. The advent of universal screening programs has virtually eliminated the devastating consequences of untreated CH and lessened experience with the acute manifestations of disease and early disease treatment. Most newborns present with primary congenital hypothyroidism due to thyroid dysgenesis (85%) or dyshormonogenesis (15%) secondary to defective embryogenesis of the thyroid gland or inborn error of hormone synthesis [2]. Secondary CH results from a deficiency of TSH, which, in neonates, is most often associated with other pituitary hormone deficiencies due to a developmental anomaly of the pituitary gland.

With the advent of universal NBS across the United States as well as in most industrialized countries, infants rarely present with clinical symptoms of CH but come to medical attention after detection by an abnormal screening result. Hence, the clinical features of CH such as prolonged jaundice, large anterior and posterior fontanelles, macroglossia, goiter, and a history of poor feeding and constipation are now rarely seen [3, 4]. Generally, the clinical features of hypothyroidism are expected to resolve once the thyroid hormone levels are biochemically normalized. The acute systemic effects of CH such as lethargy, ileus, and changes in skin and hair may be variably present following birth or develop prior to disease reporting and recognition by NBS services. Indeed as was the case for the infant presented here, ileus and functional bowel obstruction may persist for weeks beyond the normalization of serologic thyroid function tests. We would like to raise awareness of the acute gastrointestinal consequences of hypothyroidism, as early detection and immediate therapy for CH have lessened the development of its clinical manifestations in the newborn population. While early treatment improves the neurodevelopmental outcome for infants with CH, the acute systemic consequences of hypothyroidism may also be present and could lead to unexpected management challenges for pediatric health care providers.

## 2. Case Presentation

The infant was a former 39-week gestational age female born weighing 2835 grams (10th–50th percentile) after cesarean section delivery for fetal distress to a mother whose prenatal course was unremarkable. Specifically, the mother did not have a history of thyroid disease. The infant required routine care in the delivery room, did well in the nursery, and was discharged home on day 3 of life tolerating full breast milk feeds, voiding and stooling normally. The primary care pediatrician received the results of the abnormal NBS on day 12 of life and referred the infant to our hospital for confirmatory testing of thyroid function.

Physical examination revealed a sleepy infant that aroused with exam. Her neurologic exam was nonfocal. Heart rate was 122, respiratory rate 31, and blood pressure 63/43 and oxygen saturation was 100% while the infant was breathing room air. Weight was 2555 grams, a decrease of nearly 10% from birth weight. The skin was yellowish, soft, and slightly dry to touch. A soft, uniform, mobile, and thyroid gland measuring 3 cm × 1.5 cm was palpable in the anterior neck. Chest exam revealed bilateral breath sounds that were clear to auscultation with a cardiac exam demonstrating a regular rate and rhythm without murmur. The abdomen was distended with visible bowel loops and decreased bowel sounds in all quadrants. The extremities had normal tone and movement and were warm and well perfused, without evidence of edema.

**Figure 1:**
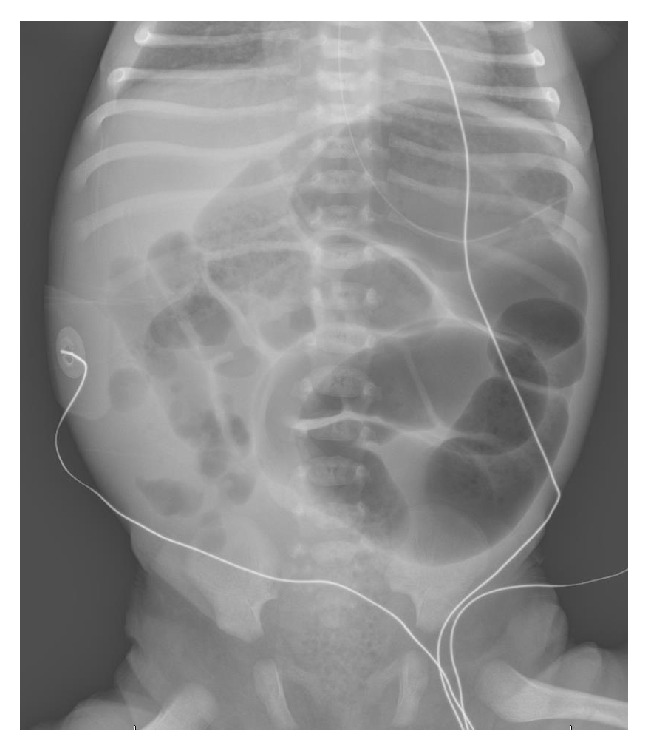
Abdominal radiograph obtained on HD 2 demonstrated dilation of the proximal bowel with normal caliber distal bowel and a paucity of air in the rectum.

Laboratory investigation revealed a thyroid stimulating hormone (TSH) level of 500 *μ*IU/mL (normal 1.8–7.97 *μ*IU/mL) and free thyroxine (FT_4_) of <0.1 ng/dL (normal 0.9–2.2 ng/dL), thus confirming primary CH. A CBC and complete metabolic panel were normal except for a total bilirubin level that was 15 mg/dL (normal < 2 mg/dL) with an unconjugated fraction of <0.1 (normal) and blood glucose of 50 mg/dL (normal > 55 mg/dL). Thyroid ultrasound revealed a diffusely enlarged thyroid gland with increased vascularity. The right lobe measured 2.8 × 1.3 × 1.3 cm and the left lobe measured 2.6 × 1.1 × 1.3 cm with 0.4 cm of thickness at the isthmus. A thyroid uptake study was consistent with a normally sited and formed thyroid gland although uptake was increased, 46% at 4 hours (normal 8–25%) and 25% uptake at 24 hours (normal 8–25%). An abdominal radiograph revealed a distended proximal bowel and normal caliber distal bowel and stool seen throughout the colon (Figure 1). Serial studies of blood glucose were consistent with borderline hypoglycemia (32–53 mg/dL) and poor feeding necessitated the transfer of the infant to the neonatal intensive care unit for further evaluation and management.

**Figure 2:**
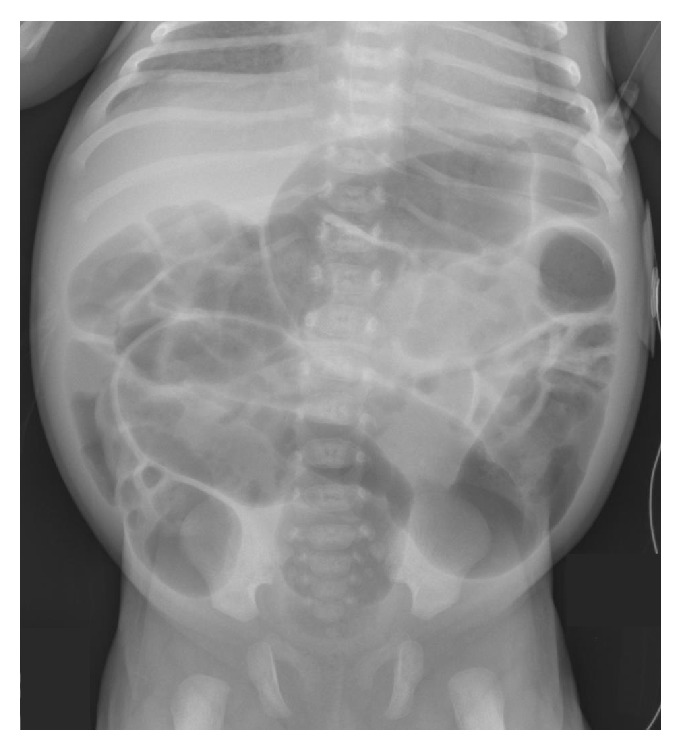
A representative abdominal radiograph obtained on HD 11 revealed gaseous distention consistent with an adynamic ileus without evidence of bowel obstruction. A paucity of air in the rectum is again seen.

**Figure 3:**
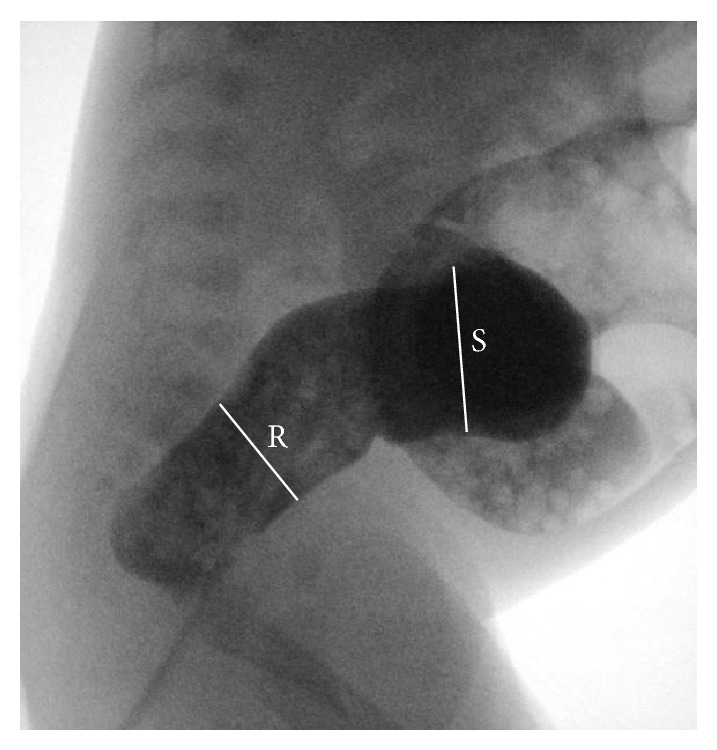
Lateral image obtained from the contrast enema demonstrated a mildly narrowed distal rectum (R) compared to a slightly larger caliber sigmoid colon (S). These results demonstrated a diminished rectosigmoid index although there was no apparent transition zone.

On hospital day (HD) 6, the TSH had decreased to 115 *μ*IU/mL and the FT_4_ had normalized to 1.6 ng/dL. On HD 12, the TSH had normalized to 10.3 *μ*IU/mL and the FT_4_ was 3.3 ng/dL. However, during these 1st two weeks of hospital stay, the infant had recurrent feeding intolerance with emesis and abdominal distention despite improvement in the thyroid function tests. The infant was made NPO on several occasions, receiving a nasogastric tube for decompression and parenteral nutrition. Enteral levothyroxine was changed to IV form. Serial abdominal radiographs continued to reveal diffusely distended loops of bowel (Figure 2) prompting a surgical consult and a recommendation for a contrast enema. The contrast enema showed no transition zone but was notable for a diminished rectosigmoid index (Figure 3) and raised concern for Hirschsprung's disease, prompting a suction rectal biopsy. The rectal biopsy revealed ganglion cells and Hirschsprung's disease was ruled out. A regimen of rectal irrigation and glycerin suppositories was initiated to foster consistent distal bowel evacuation and decompression. Expressed breast milk feeds were reinitiated on HD 14 advancing to full enteral feeds by HD 16. Parenteral thyroid hormone replacement was discontinued and alternating doses of 25 mcg and 37.5 mcg of oral levothyroxine were given. The infant was discharged home on HD 19 with twice-daily glycerin suppositories to aid bowel evacuation. The discharge weight was 3038 grams with a follow-up weight 5 days after discharge of 3227 grams (weight gain 38 grams/day). The infant was tolerating ad lib feeds and stooling consistently with normal TSH (7.23 *μ*IU/mL) and FT_4_ (1.6 nmol/L) levels.

## 3. Discussion

Poor feeding with abdominal distension is a clinical manifestation of congenital hypothyroidism; however, the severity and long duration of symptoms in our newborn, despite biochemical normalization of thyroid hormone levels, are uncommon [3, 5, 6]. Few cases are reported in the literature. Vidwans et al. and Smolkin et al. reported neonates with very similar clinical features of emesis, abdominal distension, and decreased intestinal peristalsis [5, 6]. While these infants had severe primary hypothyroidism, they responded quickly to thyroid hormone replacement and were able to tolerate full feeds allowing for discharge home within 4 and 9 days of starting thyroid hormone replacement, respectively. In a more recent case report, Sellappan et al. described a pseudoobstruction syndrome in two premature newborns that have similar responses to thyroid hormone supplementation as well [7]. In our infant, feeds were not well tolerated until 2 weeks after treatment was begun, despite rapid normalization of thyroid hormone levels. It is, however, possible that the later initiation of treatment in our case accounted for the severity and persistence of ileus.

It is unclear what other factors contributed to the delay in resolution of the gastrointestinal symptoms of hypothyroidism in our infant despite normal serum thyroid hormone levels. Based on ultrasound findings of a eutopic thyroid and increased uptake after administration of I^123^, hypothyroidism in our infant was likely secondary to dyshormonogenesis. In general, hypothyroidism in athyreosis is more severe than in dyshormonogenesis. However, it is interesting that, despite a eutopic thyroid gland, our patient had severe clinical and biochemical evidence of hypothyroidism. Abdominal distention and ileus are thought to be secondary to decreased intestinal motility, the same mechanism underlying constipation as a symptom of hypothyroidism in children and adults. Perhaps in our patient, improvement in intestinal motility required a longer recovery process related to resetting the pacemaker cells of Cajal or neuromuscular motility system in the intestine [8, 9]. In addition, although there was no thyroid dysfunction or autoimmune disease reported in the mother of our infant, an undetected thyroid disorder could have decreased thyroid hormone levels in the fetus or maternal autoantibodies could have additionally inhibited fetal thyroid function [10].

We thus report a neonate with severe congenital hypothyroidism complicated by recalcitrant ileus and abdominal distention with a prolonged recovery phase despite normalization of thyroid function soon after initiation of treatment. Through this case, we wish to raise awareness of possible uncommon clinical manifestations of congenital hypothyroidism among pediatricians, neonatologists, and endocrinologists to better enable timely and appropriate management perhaps avoiding what may be unnecessary and expensive investigations.

## Abbreviations

CH: Congenital hypothyroidism
FT_4_: Free thyroxine
HD: Hospital day
NBS: Newborn screening
TSH: Thyroid stimulating hormone.
